# Does antenatal depression predict post-partum depression and obstetric complications? Results from a longitudinal, long-term, real-world study

**DOI:** 10.3389/fpsyt.2022.1082762

**Published:** 2022-12-14

**Authors:** Mario Luciano, Matteo Di Vincenzo, Carlotta Brandi, Lucia Tretola, Rita Toricco, Francesco Perris, Antonio Volpicelli, Marco Torella, Marco La Verde, Andrea Fiorillo, Gaia Sampogna

**Affiliations:** ^1^Department of Psychiatry, University of Campania “L. Vanvitelli”, Naples, Italy; ^2^Obstetrics and Gynaecology Unit, Department of Woman, Child and General and Specialized Surgery, University of Campania “Luigi Vanvitelli”, Naples, Italy

**Keywords:** antenatal depression, perinatal, EPDS, anxiety symptoms, risk factors, gestational age, labor induction, APGAR score

## Abstract

**Background:**

Main aims of the present paper are to: (1) assess the prevalence of antenatal depression (AD) and identify its predictors; (2) analyse the impact of AD on obstetric outcomes and on the incidence of post-partum depression.

**Methods:**

All pregnant women referring to the Gynecology and Obstetrics inpatients unit of the University of Campania “Luigi Vanvitelli” were invited to participate. Upon acceptance, women completed the Italian version of the Edinburgh Postnatal Depression Scale and an *ad-hoc* questionnaire on the women's sociodemographic, gynecological and peripartum characteristics as well as their psychiatric history. Women were assessed at each trimester of pregnancy, immediately after the childbirth and after one, three, 6 and 11 months.

**Results:**

268 pregnant women were recruited, with a mean of 32.2 (±5.81) years. Ninety-seven women (36.2%) reported the presence of depressive symptoms during pregnancy. Predictors of AD were personal history of depression, a family history for depressive disorders and problematic relationships with the partner. The presence of AD was associated to a reduced gestational age at the time of delivery, a lower APGAR score at 1 and 5 min, labor induction and admission of the new-born into neonatal intensive care unit. Mothers with antenatal depression are less likely to natural breastfeed. Lastly, antenatal depression was a risk factor for higher EPDS scores at follow-ups.

**Conclusions:**

Our results support the idea that women should be screened during pregnancy and post-partum for the presence of depressive and anxiety symptoms. Health professionals should be adequately trained to detect psychiatric symptoms during pregnancy.

## Background

Several epidemiological and population-based studies have shown that women have a two-fold increased risk of developing depression compared to men (1, 2), independently from age, nationality, and other confounding factors. Several biological processes are thought to be involved in the predisposition of women to depression, including genetically determined vulnerability and hormonal fluctuations related to various aspects of reproduction. Moreover, several psychosocial events, including higher stress-reactivity, higher rates of victimization, sex-specific socialization, internalization coping style, and disadvantaged social status are all consistent contributors to the increased vulnerability of women to depression (3). The risk of developing depression is higher in certain periods of a woman's life, such as pregnancy (4) and menopause (4). In particular, during pregnancy, many relevant physiological, hormonal, social and psychological changes occur (5), which can explain why a significant proportion of pregnant women experience depressive and comorbid anxiety symptoms (6, 7). Recent reviews have reported that from 15 to 65% of women experience depressive symptoms during pregnancy, mainly characterized by depressed mood, low self-esteem, loss of appetite, feelings of fatigue and poor concentration (8). In a minority of cases, pregnant depressed women show suicidal ideation, with suicide being the leading cause of maternal deaths (9, 10). The incidence of antenatal depression is higher in low- and middle-income countries (11), which is probably due to the lack of integration between psychiatric and gynecological units, poor understanding and acceptance of mental health conditions and higher socio-economic deprivation (12). The majority of studies carried out so far have mainly focused on depressive symptoms with the onset during the post-partum period, while little attention has been devoted to antenatal depression, and to its consequences on pregnancy outcomes (13, 14). The few available studies have highlighted that antenatal depression has a negative impact on mother's future mental health and wellbeing, on the family, on the mother-baby interaction and on the long-term emotional and cognitive development of the newborn (15–17). Infant difficult temperaments and poor self-regulation, as well as increased likelihood of hospitalization and mortality, have been reported in those born by mothers affected by antenatal depression (18–20). Moreover, the presence of clinically relevant depressive symptoms is a risk factor for preterm birth and low-birth weight, infant under-nutrition and pregnancy complications, including high rates of cesarean section and induced labor (21–24).

The identified risk factors for antenatal depression include young age, low income, low educational level, history of depression and of miscarriage and pregnancy termination, history of childhood sexual abuse, concomitant high levels of anxiety during pregnancy, low self-esteem, intimate partner violence and unplanned pregnancy (5, 25, 26). However, the most recurrent and significative risk factors associated with a higher risk to develop antenatal depression are the perceived lack of social and family support (8, 27, 28) during pregnancy, which include conflicting relationships with the partner or with other family members, reduced social contacts and feelings of loneliness (29, 30). Conversely, emotional and maternal support from spouses, other family members, friends and colleagues are protective factors on maternal mental health (31), although evidence in antenatal depression is still limited. Moreover, it is worth noting that the COVID-19 pandemic is likely to increase problems related to social contact possibly increasing rates of depressive and anxiety disorders during pregnancy (32, 33). In fact, although the effects of COVID-19 pandemic on perinatal mental health are still not fully investigated, pregnant and puerperal women represent a particular vulnerable/at-risk population for developing mental health disorders, particularly during stressful situations, such as the COVID-19 pandemic. Concerns on fetal/neonatal health, transmission of COVID-19 infection from mother to fetus and worries regarding the potential separation and social distancing from family and social relationships during the perinatal period due to quarantine measures have been considered as potential risk factors for the onset of anxiety and/or depressive symptoms during pregnancy (34).

A controversial debate is the relationship between antenatal and postnatal depression. In fact, while it is reasonable that untreated depression continues to develop after the childbirth, there is limited evidence on whether the presence of antenatal depression can predict post-partum depression (25, 35). The majority of studies have assessed antenatal depression retrospectively, or have had short-term follow-ups—i.e., postnatal depression was assessed only immediately after the childbirth or after 6 weeks from delivery (36, 37). Additionally, there is not yet an agreement on the fact that the presence of antenatal depression can be considered a strong predictor of postnatal depression. In fact, Edwards et al. (13) in a sample of 431 women found that, although antenatal depression was associated with postnatal depression, more than half of the women who were depressed antenatally did not remain depressed after delivery, whereas one-third of women developed postnatal depression only after birth. Studies with long-term follow-up (i.e., up to one year after delivery) are lacking.

In this paper we report the results of a longitudinal study whose main strengths are the long-term follow-up (i.e., up to 12 months), the identification of clear predictors of antenatal depression and the risk to develop obstetric complications associated with the presentation of depressive symptoms during pregnancy. To our knowledge, these characteristics have been investigated only in a few studies.

Thus, our study aims to: (1) assess the prevalence of antenatal depression in a sample of pregnant women referring to an outpatient gynecological unit; (2) identify predictors of antenatal depression; (3) identify the impact of antenatal depression on several obstetric and fetal outcomes and on the incidence of post-partum depression.

## Methods

The study has been carried out at the Department of Psychiatry and at the Department of Gynecology and Obstetrics of the University of Campania “Luigi Vanvitelli” in Naples, Italy. All pregnant women who referred to the outpatient unit of gynecology in the period December 2019-February 2021 were invited to participate in the study. Women with a severe intellectual disability or with a pre-existing diagnosis of schizophrenia, schizoaffective disorder, delusional disorder, other not specified psychosis-spectrum disorders or with bipolar disorder were excluded. Women with a depressive episode with an onset up to 6 months prior to pregnancy were also excluded.

Upon acceptance, women were asked to complete the Italian version of the Edinburgh Postnatal Depression Scale (EPDS) (38). At baseline, the following information were collected: (1) sociodemographic characteristics (age, nationality, educational level, marital and employment status); (2) gynecological history (spontaneous or voluntary previous abortions, parity, mental disorders during previous pregnancies); (3) clinical history about current pregnancy (vaginal vs. cesarean delivery, presence of obstetric complications during pregnancy including eclampsia, gestational diabetes, epidural analgesia, episiotomy, 1 and 5 min APGAR Score and oxytocin augmentation); (4) psychiatric history (presence of pre-existing mental disorders, drug/alcohol abuse, use of psychotropic drugs); (5) social and contextual factors (relationship with the partner, family conflicts, socio-economic status, presence of life-time stressors). Women compiled the EPDS at each trimester of pregnancy, after 3 days, 1, 3, 6, and 12 months after delivery. The EPDS is the most frequently used tool to assess depressive symptoms during pregnancy and in the post-partum period, although its use after 12 months from childbirth is less common. However, we decided to use the same instrument, rather than choosing one assessment instrument more frequently used in adult depression, in order to obtain comparable data at all study timepoints.

The Edinburgh Postnatal Depression Scale (EPDS) is a 10-item self-reported screening questionnaire initially developed for use in the postnatal period to improve detection of postnatal depression. Each item is rated on a 4-point Likert scale (0–3). EPDS has satisfactory sensitivity and specificity values, and it is also sensitive to changes in the severity of depression over time. In validation studies, different cut-off points have been found. The cut-off score for severe depressive symptoms (i.e., a positive screening result) is usually ≥13 points; however, as suggested by Hewitt et al. (39), a cut-off score ≥ 10 is usually adopted for screening purposes and can be used to identify patients with major or minor depression, during the post-partum period. It must be acknowledged that the optimal EPDS cut-off scores change considerably according to cultural variations in the expression of depressive symptoms and to socio-economic variables (40, 41). Most validation studies have suggested an optimal cut off score of the EPDS ≥9 (42, 43). However, the optimal cut-off is generally lower in the postpartum than in the antepartum period (44). In this paper, we refer to an EPDS cut-off score of ≥10 as a depressive episode, recognizing that the sensitivity and specificity for a current depressive episode is >90% and >80% (45, 46), respectively.

This study has been carried out in accordance with globally accepted standards of good clinical practice, in agreement with the Declaration of Helsinki and with national and local regulations. The study investigators ensured that all involved mental health professionals are qualified and informed about the protocol and trial-related duties. The study protocol was approved by the Ethical Review Board of the University of Campania “Luigi Vanvitelli” (protocol number 98 of February 28, 2019).

### Statistical analyses

Descriptive statistics were performed for patients' socio-demographic and clinical characteristics, including the percentage of women presenting with antenatal depression. The normal distribution of outcome variables was assessed with the Kolmogorov–Smirnov test. According to the study protocol, the time points of data collection were recorded and coded using the variable “time.” The cut-off of ≥10 at EPDS was adopted to divide the sample in two groups, with or without antenatal depression. In order to identify the clinical, sociodemographic and gynecological characteristics more frequently associated with antenatal depression, chi-square analyses for categorical variables and two-tailed *t*-test analyses for continuous variables were performed to compare women with and without symptoms of antenatal depression. A linear regression model was carried out to identify risk factors for antenatal depression, using the EPDS total score as dependent variable. All variables that were statistically significant at the univariate analyses were entered as independent variables in the multivariate analyses.

A series of linear and logistic regression models were carried out to test the hypothesis that the presence of antenatal depression is a predictor of several obstetric complications and of breastfeeding. The dependent variables included in the logistic regression were: type of delivery (vaginal delivery vs. cesarean section), need for labor induction, need to admit the new-born in intensive care units and mother's willingness to breastfeed; the independent variables were duration of labor, baby gestational age (in weeks) at the time of delivery, and 1 and 5 min APGAR scores. Both logistic and regression analyses were corrected for those factors correlated with obstetric outcomes in the current literature, including mother's age, smoking during pregnancy, parity, body mass index and occurrence of complications during pregnancy.

To test the hypothesis that the presence of antenatal depression could affect the EPDS mean scores at follow-ups, a univariate general linear regression model was performed, with the EPDS total score as dependent variable. The presence of antenatal depression was entered in the model as independent variable as well as the categorical variable “time” (including all post-partum follow-up assessments) and the interaction time^*^presence of antenatal depression. The model was adjusted for several socio-demographic, clinical and contextual characteristics, as well as for obstetric and neonatal outcomes more frequently associated with perinatal depression. Statistical analyses were performed using the Statistical Package for Social Sciences (SPSS), version 17.0. For all analyses, the level of statistical significance was set at *p* < 0.05.

## Results

A total of 268 pregnant women were recruited for the study (22 in the first trimester of pregnancy, 45 in the second trimester and 201 in the third trimester of pregnancy). Recruited women had 32.2 (±5.81) years and a good level of education (42.1% had a high school degree and 40.7% a university degree). 9.7% of them had a history of depressive disorders and 22% of anxiety disorders requiring a psychopharmacological or psychological treatment. Twenty-five percent of women had a previous spontaneous abortion. The majority of recruited women (93.6%) reported a spontaneous pregnancy and 24.2% reported the onset of complications during pregnancy, such as nausea (67.2%), risk of miscarriage (11.6%) and gestational diabetes (8.6%). 12.4% of women reported having a problematic relationship with the partner, 8.2% reported conflicts with a first-degree member other than the partner, and 11.2% of the sample experienced the death of a close relative during pregnancy. Financial difficulties were reported by 7.4% of the sample (Table 1).

**Table 1:** Socio-demographic and clinical characteristics of the sample.

	**Total Sample** **(*N* = 268)**	**EPDS >10** **(*N* = 97)**	**EPDS <10** **(*N* = 171)**
Level of education, % (*N*)			
Primary School Secondary School High School Degree	2.2 (6) 14.9 (40) 42.1 (113) 40.7 (109)	4.2 (4) 13.4 (13) 41.2 (40) 41.2 (40)	1.2 (2) 15.8 (27) 42.7 (73) 40.3 (69)
Age, M ± SD	32.24 ± 5.81	32.37 ± 5.75	32.32 ± 6.04
History of depressive disorders prior to pregnancy, yes % (*N*)	9.7 (26)	19.6 (19)	4.1 (7)^***^
History of anxiety disorders prior to pregnancy, yes % (*N*)	22.0 (59)	43.3 (42)	9.9 (17)^***^
History of eating disorders prior to pregnancy, yes % (*N*)	5.2 (14)	9.3 (9)	2.9 (5)
Previous spontaneous abortions, yes % (*N*)	25.0 (67)	25.8 (25)	24.6 (42)
Previous voluntary abortions, yes % (*N*)	5.9 (16)	8.2 (8)	4.6 (8)
Spontaneous pregnancy, yes % (*N*)	93.6 (251)	94.5 (90)	94.1 (161)
Complications during current pregnancy, yes % (*N*)	24.2 (65)	20.6 (20)	26.3 (34)
Risk of miscarriage, yes % (*N*)	11.6 (31)	20.6 (20)	6.4 (11)^**^
Hypertension in pregnancy, yes % (*N*)	3.0 (8)	4.1 (4)	2.3 (4)
Gestational diabetes, yes % (*N*)	8.6 (23)	10.3 (10)	7.6 (13)
Hemorrhage during pregnancy, yes % (*N*)	8.2 (22)	11.3 (11)	6.4 (11)
Nausea, yes, % (*N*)	67.2 (180)	77.3 (75)	61.4 (105)
Conflicts with the family, yes % (*N*)	8.2 (22)	18.6 (18)	2.3 (4)^***^
Death of a first degree relative during pregnancy, yes % (*N*)	11.2 (30)	18.6 (18)	7.0 (12)
Conflicts with partner, yes % (*N*)	12.4 (33)	21.3 (27)	3.8 (5)^***^
Financial problems during pregnancy, yes % (*N*)	7.4 (20)	15.5 (15)	2.9 (5)^*^
Separation with partner during pregnancy, yes % (*N*)	2.6 (7)	7.2 (7)	0 (0)^*^
Being victim of an assault, yes % (*N*)	3.0 (8)	6.1 (6)	1.2 (2)
Loss of job, yes % (*N*)	5.9 (16)	8.2 (8)	4.6 (8)
Support from others during pregnancy, yes % (*N*)	80.1 (217)	72.1 (70)	86.0 (147)
Support from other for the baby's care after delivery, yes % (*N*)	84.3 (226)	92.8 (90)	79.5 (136)
Depression among relatives, yes % (*N*)	19.0 (51)	36.1 (35)	9.3 (16)^**^
Having a partner with depression, yes % (*N*)	4.5 (12)	4.1 (10)	0.7 (2)^*^
Having a partner with anxiety, yes % (*N*)	6.1 (16)	10.3 (13)	1.7 (3)^**^

EPDS, Edinburgh Postnatal Depression Scale;

* *p* < .05;

** *p* < .01;

*** *p* < .0001.

### Differences between women with and without antenatal depression

**Figure 1:**
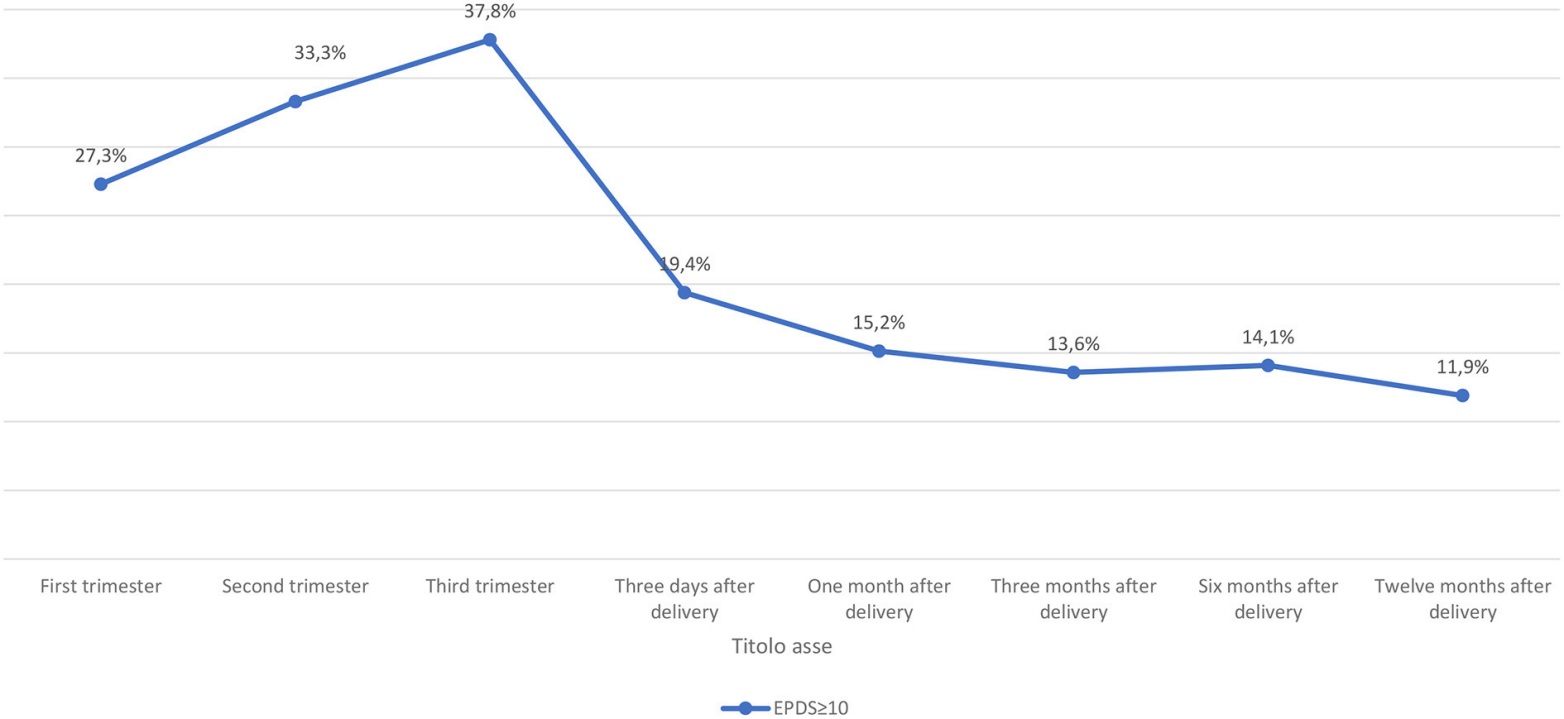
Prevalence rates of perinatal depression of women recruited during pregnancy.

Ninety-seven women (36.2%) reported a total score ≥10 at EPDS at least once during pregnancy; 27.3% (*N* = 6) screened positive at the EPDS during the first trimester, 33.3% (*N* = 15) during the second and 37.8% (*N* = 76) during the third trimester (Figure 1).

In the group with antenatal depression (AD group), the EPDS total score was 13.08 (±5.4) vs. 3.73 (±2.41) in the group without AD (*p* < 0.0001). All EPDS subscales were significantly higher in the AD group (anhedonia: 1.65 ± 1.42 in the AD group vs. 0.57 ± 0.90 in women without AD, *p* < 0.0001; anxiety: 6.48 ± 3.87 vs. 2.46 ± 1.87, *p* < 0.0001; depression: 3.63 ± 3.01 vs. 0.68 ± 1.21, *p* < 0.0001).

Compared to those without AD, women reporting depressive symptoms during pregnancy had more frequently a history of depressive (19.6% in the AD group vs. 4.1% in women without AD, *p* < 0.0001) and anxiety disorders (43.3 vs. 9.9%, *p* < 0.0001) requiring a pharmacological or psychological treatment before pregnancy. Moreover, women with AD reported more frequently risk of miscarriage (20.6 vs. 6.4%, *p* < 0.05), conflicts with the partner (21.3 vs. 3.8%; *p* < 0.0001) or with other family members (18.6 vs. 2.3%; *p* < 0.0001), and financial difficulties during pregnancy (15.5 vs. 2.9%; *p* < 0.05). Furthermore, women with AD more frequently had a close relative or the partner suffering from a depressive (4.1 vs. 0.7%; *p* < 0.05) or anxiety disorder (10.3 vs. 1.7%, *p* < 0.01), requiring a pharmacological treatment. All differences between the two groups are reported in Table 1.

### Predictors of antenatal depression

In the logistic regression model (Table 2), the likelihood to have antenatal depression was increased by the presence of: (1) a personal history of depression that required a psychiatric treatment which was still present 6 months before pregnancy (OR: 5.14; 95% CI: 2.05–8.23, *p* < 0.001); (2) a family history of depressive disorders (OR 2.10; 95% CI:0.12–4.09, *p* < 0.05); (3) a problematic relationship with the partner (OR 3.69; 95% CI: 1.20–6.18, *p* < 0.001).

**Table 2:** Predictors of antenatal depression.

			**95% Confidence interval**	
	**OR**	***p*-value**	**Lower bound**	**Upper bound**
History of depressive disorders prior to pregnancy, yes	5.14	<0.001	2.05	8.23
History of anxiety disorders prior to pregnancy, yes	1.77	0.124	−0.49	4.03
Family history for depressive disorders, yes	2.10	<0.05	0.12	4.09
Risk of miscarriage, yes	0.68	0.55	−1.55	2.91
Conflicts with relatives other than partner, yes	1.46	0.30	−1.34	4.26
Conflicts with partner, yes	3.69	<0.001	1.20	6.18
Financial problems during pregnancy, yes	1.31	0.42	−1.92	4.54
Having a partner with a depressive disorder, yes	−0.23	0.93	−5.15	4.69
Having a partner with an anxiety disorder, yes	1.47	0.388	−1.88	4.82
Constant	6.57	<0.0001	5.68	7.45

OR, Odd Ratio.

### Longitudinal assessments

Of the 268 recruited women, 165 were reassessed 3 days after childbirth and after 1 month, 162 after 3 months, 113 after 6 months and 92 after 12 months. The overall retention rate at 12-months follow up was 34%. Women were considered “drop-outs” if they did not answer to three consecutive phone calls (*N* = 110; 62.5%). Other drop-out reasons included lack of time (*N* = 50; 28.4%) and lack of interest (*N* = 16; *N* = 9.1%). No statistically significant differences were found between women who completed the 12-month follow-up assessments and those who did not in terms of age, BMI prior to pregnancy, EPDS total score at recruitment, history of depressive and anxiety disorders, type of delivery and obstetric complications during pregnancy.

According to EPDS total score, the prevalence of post-partum depression was 19% (*N* = 32) three days after delivery, 15% (*N* = 25) after 1 month, 14% (*N* = 22) after 3 months, 14% (*N* = 16) after 6 months and 12% (*N* = 11) 12 months after delivery (Figure 1).

The linear regression models (Tables 3, 4) show that the presence of antenatal depression is associated with an increased probability to have a reduced gestational age at the time of delivery (OR = 0.85, 95% CI: −1.25–2.96; *p* < 0.01), and a lower APGAR score at minutes 1 (OR = 0.81, 95% CI: 0.08–0.53; *p* < 0.05) and 5 (OR = 0.53, 95% CI: −0.05–1.15; *p* < 0.05). Logistic regression analyses also revealed that antenatal depression is a risk factor for labor induction (OR = 6.69; 95% CI: 2.51–17.83; *p* < 0.0001) and for admission of the new-born into neonatal intensive care units (OR = 1.35, 95% CI: 0.28–6.55; *p* < 0.05). Moreover, mothers with antenatal depression are less likely to natural breastfeed (OR = 1.92; 95% CI: 0.36–2.37, *p* < 0.01). These findings remained stable after controlling for several factors influencing obstetric outcomes, such as mothers' age, smoking during pregnancy, parity and body mass index.

**Table 3:** Linear regression analyses.

**Dependent variable**			**95% Confidence interval**	
	**OR**	***p*-value**	**Lower bound**	**Upper bound**
Duration of labor	1.9	NS	−0.76	4.58
Gestational age at the time of delivery (weeks)	0.85	<0.01	−1.25	2.96
APGAR score (1st min)	0.81	<0.05	0.08	1.53
APGAR score (5th min)	0.53	<0.05	−0.05	1.15

Regression analyses are corrected for mother's age, smoking during pregnancy, parity and body mass index.

**Table 4:** Logistic regression analyses.

**Dependent variable**			**95% Confidence interval**	
	**OR**	***p*-value**	**Lower bound**	**Upper bound**
Cesarean section	0.771	NS	0.326	1.82
Induction of labor	6.69	<0.000	2.51	17.83
Child in neonatal intensive care unit	1.35	<0.05	0.28	6.55
Mothers' willingness to breastfeed	1.92	<0.01	0.36	2.37

Regression analyses are corrected for mother's age, smoking during pregnancy, parity and body mass index.

### Predictors of post-natal depression

According to the regression model (Table 5), antenatal depression is a risk factor for higher EPDS scores at follow-ups (OR = 3.45; 95% CI: 6.87–8.61; *p* < 0.001), remaining stable after controlling for several sociodemographic, clinical, contextual and gynecological variables. Moreover, when exploring the interaction time^*^presence of antenatal depression, the risk is almost three times higher 3 days (OR = 3.44; 95% CI: −1.04–7.93; *p* < 0.001), 1 month (OR= 3.09; 95% CI: −1.37–7.54; *p* < 0.05), and 6 months (OR = 3.53; 95% CI: −1.00–8.06; *p* < 0.01) after delivery. Other risk factors include the presence of a depressive disorder requiring a psychiatric treatment before pregnancy (OR = 2.96, 95% CI: 1.11–4.81, *p* < 0.01), family conflicts (OR: 1.78; 95% CI: 0.38–5.18, *p* < 0.05) and financial difficulties (OR: 1.59; 95% CI: −0.30–3.47, *p* < 0.05).

**Table 5:** Predictors of postnatal depression at follow-ups (dependent variable: EPDS main score).

			**95% Confidence interval**	
	**OR**	***P*-value**	**Lower bound**	**Upper bound**
Intercept	3.95	<0.05	0.31	7.58
History of depressive disorder prior of pregnancy, yes	2.96	<0.01	1.11	4.81
History of anxiety disorders prior to pregnancy, yes	0.17	NS	−1.16	1.50
Risk of miscarriage, yes	0.65	NS	−1.89	3.13
Having a partner with depression, yes	−0.49	NS	−3.04	2.05
Having a partner with an anxiety disorder, yes	−0.23	NS	−1.97	1.51
Family conflicts, yes	2.78	<0.05	0.38	5.18
Conflicts with partner, yes	0.12	NS	−1.37	1.60
Financial problems, yes	1.59	<0.05	−0.30	3.47
Presence of antenatal depression	3.45	<0.001	6.87	8.61
Time*presence of antenatal depression				
Three days after delivery	3.44	<0.001	−1.04	7.93
One month after delivery	3.09	<0.05	−1.37	7.54
Three months after delivery	1.08	NS	−3.49	5.65
Six months after delivery	3.53	<0.01	−1.00	8.06
Twelve months after delivery	3.15	NS	−2.51	8.80

## Discussion

In this study we explored factors influencing the presence of antenatal depression and whether the presence of antenatal depression has an impact on obstetric complications during the childbirth and on the onset of depressive symptoms up to 12 months after delivery. To the best of our knowledge, this is the first study on the impact of AD on the risk to develop depressive symptoms up to 12 months after delivery in a sample of Italian women. Although antenatal depression has been less frequently studied compared to post-partum depression, there is now an increased awareness of AD adverse effects on obstetric outcomes and on baby's mental and physical health.

Thirty-six percent of women had clinically relevant symptoms of antenatal depression, with a peak in the third trimester. In other studies (47), the rates of antenatal depression ranged between 15 and 65%, with higher rates found in low- and middle income-countries. In another study carried out in Italy (48), the global prevalence rate of antenatal depression was similar to ours (31.1%). These discrepancies may be partially due to the assessment instruments adopted in the different studies. In our study depressive symptoms were investigated with the EPDS, which is the recommended screening tool for depressive symptoms during the perinatal period; other studies have used more generic assessment instruments, such as the Beck Depression Inventory (BDI), the Center for Epidemiological Studies Depression Scale (CES-D) or the Hamilton Rating Scale for Depression. However, although these tools are widely used in clinical practice, they are not appropriate for screening purposes. Moreover, even when studies have used the EPDS, different cut-offs were considered. Generally, a cut-off of 10 or more is considered adequate to include most women with mild to severe depressive symptoms, whereas some studies have chosen a cut-off of 9, and others have used more restrictive criteria (EPDS > 12) to reduce the possibility of false positives. However, in all the few available studies, women have not been assessed in the three trimesters of pregnancy, but mostly in the third trimester, where the prevalence of AD is consistently higher.

Concerning predictors of antenatal depression, the likelihood to suffer from antenatal depression is higher in women with a previous depressive episode (OR = 5.14), in those with a family history of depressive disorders (OR = 2.10) and in those with a problematic relationship with the partner (OR 3.69). Women with a personal or family history of depression have a ten-fold increased risk to develop AD (49), which may be due to the presence of a genetic vulnerability to depression, which is triggered by hormonal, behavioral, lifestyle and body changes that occur during pregnancy. Moreover, depressed women who stop taking antidepressants at the beginning of pregnancy, due to fear of teratogenicity and side effects, may have the onset of a new depressive episode during pregnancy. Another predictor of antenatal depression is the presence of an unstable relationship with the partner. This finding, which is in line with available literature, confirms that social and professional support for pregnant women plays a significant role in the prevention of antenatal depression. In particular, low social support during pregnancy, and specifically lack of support from the partner, is associated with the onset of anxiety and depressive symptoms, feelings of loneliness and hopelessness (50). On the contrary, supportive partners help pregnant women to moderate stress related to pregnancy, increase their feelings of self-efficacy (51), and improve problem-solving and coping skills (52).

We found that several obstetric complications, including preterm birth, a reduced 1- and 5-min APGAR scores, the pharmacological induction of labor and the child admission in neonatal intensive care unit, are associated with AD. The causal association between AD and adverse gynecological outcomes is already established, due to several biological and psychosocial factors. In particular, antenatal depression, associated with chronic stress and hormonal changes, may lead to hypothalamic-pituitary dysfunctions, resulting in a higher secretion and release of cortisol, with a reduced perfusion of placenta and reduced flow of oxygen and nutrients to the fetus (31). Moreover, mothers with AD may have reduced access to maternal health services, with reduced physical check-ups, and unhealthy lifestyle behaviors (24), such as poor dietary habits, smoking during pregnancy, alcohol, and substance abuse (53–55), which could lead to a reduced fetal intrauterine growth and explain some of the adverse obstetric complications found in AD patients.

Another interesting finding is that women with antenatal depression are less likely to engage in breastfeeding, whose importance for infant health is widely recognized (56). This result is rather new, since only a few observational studies have explored factors influencing the likelihood of mothers with AD to breast feed. According to Gagliardi et al. (56), mothers with higher EPDS scores immediately after delivery are more likely to bottle feed at 3 months, and that even mild depressive symptoms are associated with a dose-dependent increase in the risk of unsuccessful breast-feeding. It may be that women with AD perceive the breast feeding more stressful because of reduced energy, fatigue and abulia associated with depression. This result further supports the idea that women with antenatal depression should be supported with adequate and specific interventions, which should include a motivational approach dedicated to breastfeeding (57).

In our sample, women with AD are more likely to develop post-partum depression. Moreover, women with post-partum depression present a series of other risk factors, including financial difficulties, lack of social support from other family members and a personal history of depression. These results highlight that antenatal depression can be considered a precursor of post-natal depression and support the notion that women with a personal vulnerability to depression, who are not adequately supported and that have to face unstable socio-economic conditions, are at higher risk to develop depressive symptoms not only during pregnancy, but during the entire perinatal period (58).

Our study has several important strengths. First, its longitudinal design. In fact, the bulk of evidence on perinatal depression comes from screening studies which, despite including adequate sample sizes, assessed women only once during pregnancy or in the post-partum period. The longitudinal design of our study allowed us to identify cause-effect relationships between gynecological and psychiatric factors and to identify predictors and moderators of gynecological outcomes at delivery. Moreover, the few longitudinal available studies had a short-term follow-up (i.e., women were assessed up to 3 months after delivery). On the contrary, in our study we assessed women from the first trimester of pregnancy up to 1 year after delivery, which allowed us to detect the onset of depressive symptoms at any time during the perinatal period, to follow-up women with depressive symptoms for a long time, to assess the correlation between antenatal and postnatal depression and to assess differences in the onset of depressive symptoms in the whole perinatal period. Moreover, women with depressive symptoms with onset up to 6 months before pregnancy or with pre-existing major psychiatric disorders (schizophrenia or other primary psychoses and bipolar disorders) were excluded from the study. Even if this choice may have reduced the sample size, it allowed us to detect only new cases of depressive symptoms during the perinatal period. This is one of the major strengths of the study, since in previous studies only rarely already depressed participants were excluded from recruitment.

However, our study has obviously some limitations. First, the small sample size, since perinatal depression is not highly prevalent in the general population, large sample sizes are needed to perform appropriate analyses and draw firm conclusions. Another limitation is the fact that only 92 out of 268 women recruited during pregnancy compiled the EPDS at 1 year, with a retention rate of 34.32%. However, this high dropout rate is similar to other longitudinal studies carried out with similar methodologies (59, 60), and many other studies have adopted shorter follow-ups. Another possible limitation is that the presence of a history of anxiety and/or depressive symptoms in mothers and their relatives was evaluated only retrospectively; we have tried to limit this recall bias by considering only those affective and anxiety episodes that needed a psychiatric or psychological treatment. Moreover, we must acknowledge that the EPDS, despite being the most adopted assessment tool during the perinatal period and being associated to a satisfactorily sensitivity, should not be considered a diagnostic tool and a diagnosis of major depression should be confirmed through a clinical assessment. Furthermore, EPDS is a self-reported assessment instrument, and therefore results can be biased by the fact that women could feel not confident in reporting depressive symptoms, especially after the childbirth, due to stigma and prejudices. Lastly, the majority of women have been recruited during the third trimester of pregnancy, reducing the generalizability of our findings.

## Conclusions

In conclusion, our results support the idea that women should be screened during pregnancy and post-partum for the presence of depressive and anxiety symptoms. Our results have important clinical and research implications. First, most women with antenatal or postnatal depression do not seek help for their depressive symptoms and professionals are often not adequately trained to detect and treat them. Second, depressive symptoms during pregnancy can have serious consequences also on child affective and cognitive development. Third, our data support the need to identify new strategies to adequately treat this serious mental health condition and reduce the long-term negative impact on the mothers as well as on their babies and family members.

Health professionals working in maternal health units should be adequately trained to detect psychiatric symptoms in pregnant women. By using the new technologies (61–63), as learnt during the recent COVID-19 pandemic (64, 65), antenatal depression may be easily screened on web-based platforms (66), which are associated with a higher engagement with health care services.

Moreover, maternal and mental health services should increase collaboration to implement dedicated pathways to care in order to provide an integrated support for women at risk to develop depressive symptoms during the whole perinatal period.

## Data availability statement

The raw data supporting the conclusions of this article will be made available by the authors, without undue reservation.

## Ethics statement

The study protocol was approved by the Ethical Review Board of the University of Campania Luigi Vanvitelli (protocol number 98 of February 28, 2019). The patients/participants provided their written informed consent to participate in this study.

## Author contributions

ML, AF, GS, and MT contributed to the conceptualization and to the administration of the project, to the development of the study methodology, and to the writing of the original draft. ML, GS, MLV, and MD carried out formal analyses. CB, LT, RT, FP, and AV carried out investigations. All authors contributed to the review and revision of the final version of the manuscript.
